# A Case of Bradycardia-Induced Torsades De Pointes in a Patient Presenting to the Emergency Room With Cardiac Arrest

**DOI:** 10.7759/cureus.46831

**Published:** 2023-10-11

**Authors:** Dibyasundar Mahanta, Ramachandra Barik, Anup K Budhia, Debasish Das, Debasis Acharya

**Affiliations:** 1 Cardiology, Siksha 'O' Anusandhan (SOA) Institute of Medical Sciences (IMS) SUM Medical College and Hospital, Bhubaneswar, IND; 2 Cardiology, All India Institute of Medical Sciences, Bhubaneswar, IND; 3 Medicine, Hi-Tech Medical College and Hospital, Bhubaneswar, IND; 4 Cardiology, Hi-Tech Medical College and Hospital, Bhubaneswar, IND

**Keywords:** bazett’s formula, isoprenaline, temporary pacemaker, torsades de pointes, qtc interval, polymorphic ventricular tachycardia

## Abstract

Torsades de pointes (TdP) is a less common type of ventricular tachycardia (VT) characterized by polymorphic VT of changing amplitude and characteristic twists around the isoelectric baseline. It is almost always associated with QT interval prolongation. Unless immediately intervened, it can lead to ventricular fibrillation followed by cardiac arrest. We report a case of a patient with bradycardia-induced TdP who presented to the emergency room with cardiac arrest.

## Introduction

Polymorphic ventricular tachycardia (VT) in the setting of prolonged QT is called torsades de pointes (TdP), which is characterized by changing amplitude and twisting around the isoelectric baseline [1]. Long QT syndrome (LQTS) can be either inherited or acquired and can lead to a potentially life-threatening arrhythmia caused by early depolarization. Acquired LQTS can arise from various factors, including using QT-prolonging medications, electrolyte imbalances, acute coronary ischemia, or bradycardia. Sometimes, prolonged QT may result from bradycardia induced by atrioventricular (AV) block. It is imperative to note that patients with AV block, prolonged QT, and R-on-T phenomenon are at a high risk of developing life-threatening TdP. This could ultimately lead to ventricular fibrillation, cardiac arrest, and death [2].

## Case presentation

A 65-year-old male with no history of illness or medication intake and no family history of sudden cardiac arrest presented to the emergency room with multiple syncopal episodes over eight hours before presentation. He was taken to a local primary health center and referred to a higher center for evaluation and management. During the hospital transfer in an ambulance, he suddenly collapsed and lost consciousness. His son noticed no spontaneous breathing and no pulse. He was given chest compressions, followed by rescue breathing by his son, and was revived. Just after reaching the emergency department, he again collapsed. The monitor showed a flatline (asystole), indicating cardiac arrest. The emergency team immediately jumped into action. The patient received high-quality cardiopulmonary resuscitation (CPR) as per guidelines. Airways were managed using a bag-valve mask to ventilate.

One mg of epinephrine was given intravenously. After two rounds of CPR, the return of spontaneous circulation (ROSC) was achieved. Post-cardiac arrest, he was stabilized and closely monitored for any changes in vitals. On examination, the pulse rate was 52 per minute and was pulsus bigeminus in character. The blood pressure in the right arm (supine position) was 140/90 mm of Hg, and the respiratory rate was 30/min. The respiratory system and cardiovascular system examinations were within normal limits. He was confused for 15 minutes after CPR. A neurological system examination after 15 minutes and subsequently after a few hours revealed no abnormality. Immediately, an electrocardiogram (ECG) was taken, and blood samples were drawn to assess electrolyte levels, arterial blood gases (ABGs), and other relevant markers. The ABG levels were within the normal range. His blood sugar was 119 mg/dl. The high-sensitive cardiac troponin T (hs-cTnT) assay was done twice. The first was done just after resuscitation for cardiac arrest in the emergency department, and the second was done on the second day. The levels of hs-cTnT were 9 ng/L and 13 ng/L, respectively (normal range: 0-14 ng/L). As his hs-cTnT levels were within the normal range twice and the patient had no symptoms of acute coronary syndrome (ACS), it was ruled out, and a coronary angiogram (CAG) was not done.

**Figure 1:**
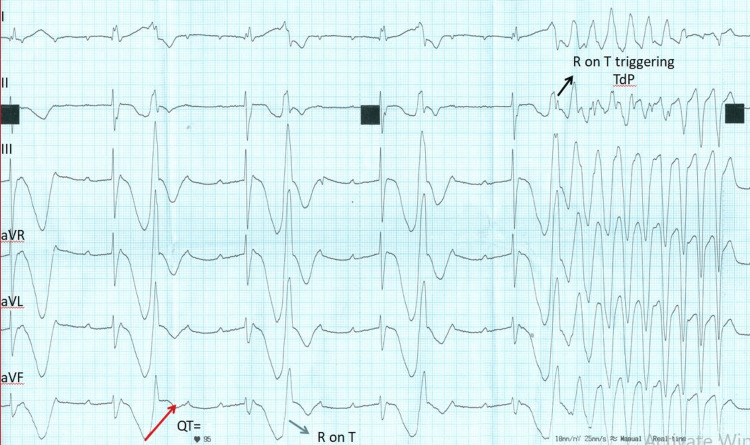
Post-CPR six-lead ECG at the speed of 25 mm/sec An ECG showing AV dissociation suggestive of complete heart block, QT= 1000 msec, was calculated after extrapolating the terminal portion of the T wave towards the baseline (red arrow). Immediately after the RBBB escape complex, a short coupled ectopic ventricular complex falls on the T wave (the R on T phenomenon indicated by the blue arrow) in the bigeminal pattern. Towards the later part of the ECG, R-on-T initiates Torsades de Pointes (black arrow). CPR: cardiopulmonary resuscitation; ECG: electrocardiogram; AV: atrioventricular; RBBB: right bundle branch block

The ECG showed a ventricular bigeminy pattern, a heart rate of 52 per minute, and complete atrioventricular (AV) dissociation (Figure 1).

The right bundle branch block (RBBB) morphology escape focus was followed immediately by a short coupled ventricular ectopic falling on the T wave of the preceding escape beat. If we closely observe and extrapolate the terminal portion of the T wave towards the baseline (red arrow in Figure 1), then QT is found to be prolonged (QT=1000 mec). Using the Bazett formula, QTc was 931 msec (Bazett formula: QT/√RR) [3]. A short coupled ectopic R wave is falling on the terminal portion of the T wave (the R on T phenomenon shown by the blue arrow). Towards the end of the ECG strip, the R-on-T triggers a polymorphic ventricular tachycardia (black arrow) twisting around the baseline. This specific pattern of VT with QT prolongation is called torsades de pointes. So, a third-degree AV block is associated with QT prolongation and the R-on-T phenomenon triggering TdP.

**Figure 2:**
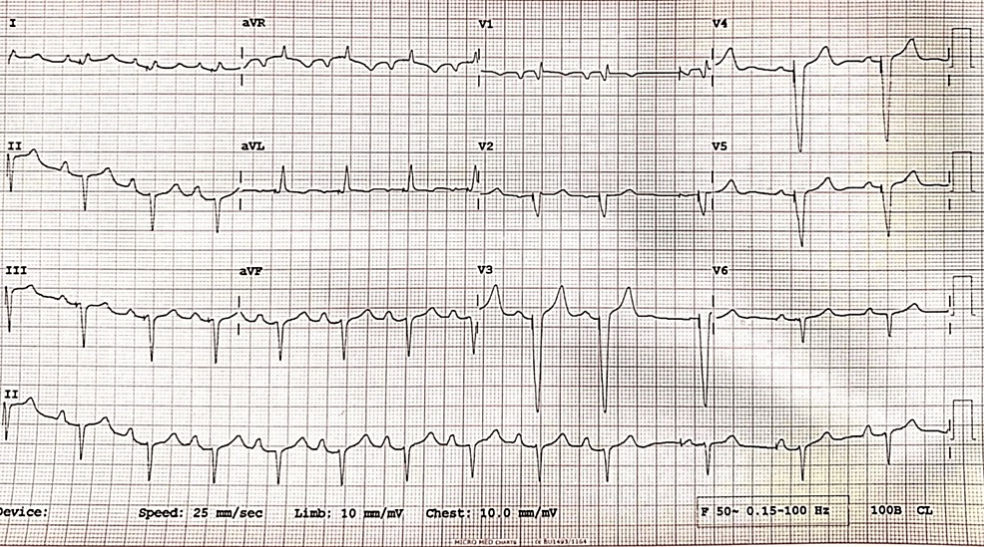
The post-implantation ECG shows AsVp and ApVp and a QTc of 434 msec. AsVp: atrium sense, ventricle pace; ApVp: atrium pace, ventricle pace; QTc: QT corrected for heart rate

As VT was nonsustained, an immediate isoprenaline infusion started to increase the heart rate. The patient was shifted immediately to the cath lab for temporary pacemaker insertion and paced at a rate of 100/min. At a pacing rate of 100/min, QTc decreased, the R-on-T phenomenon disappeared, and no further VT appeared. Serum electrolytes were as follows: sodium: 139 mEq/dL, potassium: 4.5 mEq/dL, calcium: 9.1 mEq/dL, phosphate: 3.2 mEq/dL, and magnesium: 1.9 mEq/dL. The echocardiogram showed a structurally normal heart. A dual-chamber rate-modulated (DDDR) was implanted electively with suitable sensing and pacing parameters. Post-implantation ECG shows atrium sense, ventricle pace (AsVp), atrium pace, ventricle pace (ApVp), and a QT corrected for heart rate (QTc) of 434 msec (Figure 2).

The patient was discharged in stable condition after five days.

## Discussion

Many clinical situations may cause acquired QT prolongation, which is a risk factor for ventricular arrhythmias and sudden cardiac death. Severe bradycardia or AV block, QT-prolonging drugs, dyselectrolytemia, and acute ischemia are important causes of acquired LQTS. In these patients, the R-on-T phenomenon generally precedes the initiation of ventricular arrhythmia. Smirk first described the R-on-T phenomenon in 1949 [1].

In 1966, Francois Dessertenne described a specific electrocardiographic form of polymorphic ventricular tachycardia (PVT) characterized by changing amplitude with a characteristic twist around the isoelectric baseline with a prolonged QT interval, which he termed “torsades de pointes” [2]. In acquired long QT, bradycardia or pause caused by short, long, short cycles typically precede classical TdP, hence the term pause-dependent. In contrast, in the case of congenital prolonged QT, it is not pause-dependent [4]. The QT interval represents the repolarization part of a cardiac cycle and the most vulnerable part of a cardiac cycle. The QT prolongation increases the repolarization period. Dyselectrolytemia and QT-prolonging drugs increase repolarization by blocking IKr (the rapid rectifying potassium channel). As ion channel expression differs in different regions of the ventricular myocardium and dyselectrolytemia and QT-prolonging drugs affect particular ion channels, a repolarization gradient is formed. If the gradient is sufficient, it triggers an action potential, which further triggers more action potential to cause ventricular arrhythmia. Some conditions, like Brugada syndrome, hypertrophic cardiomyopathy, acute myocardial ischemia, and catecholaminergic ventricular tachycardia (CPVT), can give rise to the R-on-T phenomenon and polymorphic VT without QTc prolongation.

Withdrawal of QTc-prolonging drugs, treatment of dyselectrolytemia, isoprenaline infusion, and temporary pacemaker insertion to increase the heart rate are treatment options for R-on-T with prolonged QTc-causing TdP [5]. Amiodarone is contraindicated in this case because it can further increase QTc. Amiodarone infusion can be given in the case of R on T with normal QTc, causing polymorphic VT. A heart rate below 60 beats per minute has the greater potential to develop R-on-T with prolonged QT and TdP, according to Kurita et al. [6]. A QT interval above 600 msec in a patient with AV block increases the risk of TdP [6].

## Conclusions

To conclude, some patients with severe bradycardia or AV block are associated with QT prolongation and are vulnerable to episodes of life-threatening TdP resulting in syncope, cardiac arrest, and death due to ventricular fibrillation. Early recognition of this high-risk feature in the ECG and urgent implantation of a temporary pacemaker can be lifesaving.
